# Genome-Wide Identification and Expression Profiling of HD-Zip Family Genes in Flax (*Linum usitatissimum* L.)

**DOI:** 10.3390/cimb48040402

**Published:** 2026-04-14

**Authors:** Yamin Niu, Yanni Qi, Limin Wang, Wenjuan Li, Zhao Dang, Yaping Xie, Wei Zhao, Gang Wang, Zuyu Hu, Nan Lu, Xiaoyan Zhu, Jing Zheng, Junyan Wu, Jianping Zhang

**Affiliations:** 1 College of Agriculture, Gansu Agricultural University, Lanzhou 730070, China; 1073324120574@st.gsau.edu.cn (Y.N.); 1073324120594@st.gsau.edu.cn (Z.H.); 1073324120393@st.gsau.edu.cn (N.L.); 1073325020210@st.gsau.edu.cn (X.Z.); 1073325120503@st.gsau.edu.cn (J.Z.); wujuny@gsau.edu.cn (J.W.); 2 Institute of Crop, Gansu Academy of Agricultural Sciences, Lanzhou 730070, China; xbsdqyn@126.com (Y.Q.); liminwang@aliyun.com (L.W.); liwenjuan@gsagr.ac.cn (W.L.); dangzhao@gsagr.cn (Z.D.); xieyp2012@126.com (Y.X.); zhaowei@gsagr.ac.cn (W.Z.); zyyw1216@aliyun.com (G.W.)

**Keywords:** flax, *HD-Zip* gene family, hormone response, abiotic stress, gene

## Abstract

The homeodomain-leucine zipper (HD-Zip) transcription factor family is conserved in land plants and is critical for regulating growth, development, and stress responses. Flax (*Linum usitatissimum* L.) is an economically valuable dual-purpose crop valued for its high nutrition and notable drought tolerance; however, its *HD-Zip* gene family has not been systematically characterized. In this study, a comprehensive genome-wide analysis was performed to identify and characterize the *HD-Zip* family in flax. A total of 34 *LuHD-Zip* genes were identified, which were unevenly distributed across 15 chromosomes and exhibited substantial variation in physicochemical properties. The encoded proteins ranged from 200 to 372 amino acids in length, with molecular weights of 22.7–40.3 kDa and theoretical isoelectric points (pI) of 4.49–9.46. All LuHD-Zip proteins were predicted to be hydrophilic and localized to the nucleus. Phylogenetic analysis divided these proteins into two major subfamilies (Group 1 and Group 2), a classification strongly supported by conserved gene structures and motif compositions, implying potential functional redundancy within each group. Gene duplication analysis revealed that segmental duplication events (29 pairs) were the primary drivers of family expansion. Comparative syntenic analysis further indicated that the *LuHD-Zip* gene family has remained relatively conserved throughout evolution. Promoter cis-element analysis identified multiple regulatory elements associated with hormone signaling and abiotic stress responses, suggesting complex transcriptional control in response to environmental stimuli. Expression profiling via quantitative real-time PCR (qRT-PCR) demonstrated that *LuHD-Zip* genes exhibit tissue-specific expression patterns and are differentially regulated by various phytohormone treatments and abiotic stresses. This study provides the first genome-wide characterization of the *HD-Zip* gene family in flax, offering valuable insights into its evolution and potential functions. These findings establish a solid foundation for future functional investigations of the *LuHD-Zip* gene family.

## 1. Introduction

Flax (*Linum usitatissimum* L.), a dicotyledonous plant, is a member of the family Linaceae [1]. As an ancient dual-purpose crop cultivated for both its fibers and oil, flax is widely grown around the world, particularly in major producing regions such as China, Canada, France, Russia, Ukraine, the United States, and Argentina [2]. As a significant economic crop, flax holds a prominent position among oilseed crops due to its exceptional nutritional profile and versatile applications. Beyond its traditional use in natural fiber and oilseed production, flax has gained recognition as a functional food for its substantial health benefits. It serves as a valuable source of oil with significant health-promoting properties, while also being extensively utilized in the manufacturing of industrial products such as drying oils, pigments, textiles, linoleum, plastics, and printing inks. Furthermore, flax has found applications in therapeutic uses, the energy sector, and as a source of crude fiber in animal nutrition [3]. In addition, flax is one of the most important industrial crops cultivated globally and is suited to various habitats and agro-ecological conditions. However, flax plants encounter numerous abiotic and biotic stressors due to environmental changes, which can directly or indirectly affect plant health [4]. Therefore, in-depth exploration of stress-responsive transcription factor genes in flax is of great significance for the genetic improvement of flax and for advancing stress-resistant breeding in oilseed crops.

Transcription factors (TFs) are DNA-binding proteins that specifically recognize and interact with cis-regulatory elements in the promoter regions of target genes. By modulating gene expression patterns, they play a central role in plant growth, development, cellular differentiation, and adaptation to environmental cues [5]. The homeodomain-leucine zipper (*HD-Zip*) gene family encodes a class of transcription factors unique to higher plants and plays a vital role in regulating plant growth, development, environmental adaptation, and stress responses [6]. Members of this family have been widely identified in various plant species, including *Arabidopsis thaliana*, rice (*Oryza sativa*), maize (*Zea mays*), soybean (*Glycine max*), and potato (*Solanum tuberosum*) [7,8,9,10,11,12]. A distinctive structural feature of HD-Zip proteins is the presence of two conserved domains: a homeodomain (HD) and a leucine zipper (LZ) motif [13]. The HD comprises approximately 60 highly conserved amino acid residues responsible for sequence-specific binding to target DNA, while the LZ domain contains 35–42 amino acid residues and mediates protein dimerization [14]. Based on sequence conservation, protein function, gene structure, and other characteristics, the HD-Zip family can be classified into four subfamilies: HD-Zip I-IV [6]. Functional studies have further revealed distinct roles for each HD-Zip subfamily in plant growth and development. For instance, HD-Zip I proteins are actively involved in plant responses to abiotic stress [15]. HD-Zip II proteins, such as *Athb-2* and *Athb-4*, have been demonstrated to play a critical role in promoting shade avoidance responses in many angiosperms [16]. In the root apical meristem, the periclinal division of vascular bundle cells is regulated by plant hormones and key transcription factors, including HD-Zip III family proteins, which have been shown to positively regulate brassinosteroid biosynthesis-related genes in vascular tissues [17]. Previous studies have shown that many HD-Zip IV proteins are primarily involved in regulating anthocyanin accumulation, cell differentiation, root development, and trichome formation [6,13,18]. For instance, *AtML1* and *AtPDF2* play roles in regulating shoot epidermal cell differentiation in *Arabidopsis*, whereas *AtGL2* and *AtHB10* negatively regulate hair formation, playing a key role in trichome and root hair development [14,19].

The availability of high-quality genomic data for flax has advanced considerably with the development of high-throughput sequencing technologies, offering a robust foundation for exploring gene family functions and regulatory networks. However, the *HD-Zip* gene family in flax has not yet been systematically characterized. In particular, the identification of its members, their evolutionary relationships, expression patterns, and potential roles in growth, development, and stress responses remain largely unexplored. In this study, we performed a genome-wide identification and comprehensive analysis of the *HD-Zip* gene family in flax. Using integrated bioinformatic approaches, we characterized their physicochemical properties, chromosomal distribution, gene duplication events, phylogenetic relationships, gene structures, conserved motifs, promoter cis-elements, and expression profiles. Our findings provide valuable insights into the potential functions of *HD-Zip* genes in flax.

## 2. Materials and Methods

### 2.1. Plant Materials and Stress Treatments

The flax cultivar ‘Longya-10’, bred and maintained in our laboratory, was used as the experimental material. Seeds were surface-sterilized with 70% ethanol for 30 s, followed by five rinses with sterile distilled water. The sterilized seeds were placed in Petri dishes containing moist filter paper and germinated at 25 °C. Uniformly germinated seedlings were transplanted into a growth chamber and cultivated at 25 °C with 60% relative humidity under a 16 h light/8 h dark photoperiod at a light intensity of 200 μmol·m^−2^·s^−1^. After 14 days, seedlings of uniform size (approximately 8–10 cm in height) were transferred to half-strength Murashige and Skoog (1/2 MS) liquid medium (pH 5.8; Murashige and Skoog basal medium, Sigma-Aldrich, St. Louis, MO, USA) and acclimated for 4 days under the same growth conditions [20]. For stress treatments, seedlings were transferred to fresh 1/2 MS medium supplemented with 20% (*w*/*v*) polyethylene glycol (PEG 6000, analytical grade, Sigma-Aldrich, St. Louis, MO, USA), 15 μM abscisic acid (ABA, purity ≥ 98%, Solarbio Science & Technology Co., Ltd., Beijing, China), or 10 μM methyl jasmonate (MeJA, purity ≥ 98%, Solarbio Science & Technology Co., Ltd., Beijing, China). Temperature stress was applied by placing seedlings in growth chambers set at 42 °C (heat stress) or 4 °C (cold stress), while control seedlings were maintained at 25 °C. All plants used for treatments were derived from the same sowing batch, and three independent biological replicates were performed for each treatment and each sampling time point. Leaf samples (the second and third fully expanded leaves from the apex) were collected at 0, 3, 6 and 9 h after cold and heat stress treatments, and at 0, 3, 6, 9, 12, 24, 48, and 72 h after PEG, ABA, and MeJA treatments. Following stress exposure, a subset of seedlings was transferred to stress-free 1/2 MS liquid medium and allowed to recover for 48 h under normal growth conditions (25 °C), with samples harvested at the end of the recovery period. For tissue-specific expression analysis, flax plants were grown in sterile nutrient soil consisting of peat moss, vermiculite, and perlite in a 2:1:1 ratio (*v*/*v*/*v*; nutrient content: N 1.0 g/kg, P_2_O_5_ 0.8 g/kg, K_2_O 1.2 g/kg, pH 5.5–6.5; Huawen Agricultural Technology Co., Ltd., Jinan, China) under normal growth conditions (25 °C, 60% relative humidity, 16 h light/8 h dark photoperiod, light intensity of 200 μmol·m^−2^·s^−1^) until reaching the full-bloom stage. Roots, stems, young leaves (the first fully expanded leaf from the apex), flowers (at full-bloom stage), and capsules (15 days after flowering) were separately collected from these soil-grown plants, which received no stress treatment. Soil cultivation was employed to ensure normal growth and development, enabling the collection of complete tissues—including flowers and capsules—that are difficult to obtain under MS liquid medium culture. All harvested samples were immediately frozen in liquid nitrogen and stored at −80 °C until processed for total RNA extraction and quantitative real-time reverse transcription polymerase chain reaction (qRT-PCR) analysis.

### 2.2. Identification of LuHD-Zip Gene Family

The genome sequence of flax cultivar “Longya-10” used in this study was previously assembled by our research group and has been deposited in the NCBI database under the accession number QMEI00000000. The corresponding protein dataset was obtained from the Figshare repository (https://figshare.com/; accessed on 18 December 2025). To identify HD-Zip family members, protein sequences of *HD-Zip* genes from *Arabidopsis thaliana* were retrieved from The *Arabidopsis* Information Resource (TAIR; https://www.arabidopsis.org/; accessed on 18 December 2025). Additionally, HD-Zip protein sequences from soybean (*Glycine max*) [12], maize (*Zea mays*) [21], rice (*Oryza sativa*) [22], and potato (*Solanum tuberosum*) [10] were obtained from previously published genomic datasets. These sequences were used as queries to perform BLASTP (version 2.17.0) searches against the Longya-10 protein dataset under default parameters. Candidate proteins were subsequently verified for the presence and integrity of the conserved HD-Zip domain using the NCBI Conserved Domain Database (CDD; https://www.ncbi.nlm.nih.gov/cdd/ (accessed on 19 December 2025)) and InterPro (https://www.ebi.ac.uk/interpro/ (accessed on 19 December 2025)). After manual curation, 34 non-redundant LuHD-Zip proteins were identified. For each identified protein, the molecular weight (MW), theoretical isoelectric point (pI), grand average of hydropathicity (GRAVY), and amino acid length were computed using the ExPASy ProtParam tool (https://web.expasy.org/protparam/, accessed on 20 December 2025 [23]. Conserved motifs were identified using the MEME suite (https://meme-suite.org/meme/tools/meme; accessed on 21 December 2025) with default parameters [24].

### 2.3. Chromosomal Localization and Collinearity Analysis

The chromosomal positions of the *LuHD-Zip* genes were extracted from the annotation files of the “Longya-10” genome. Their distribution along the chromosomes was visualized using the MapGene2Chromosome (MG2C) tool (v2.0; http://mg2c.iask.in/mg2c_v2.0/, accessed on 24 December 2025). Gene duplication events, including tandem and segmental duplications, were identified using the MCScanX package with default parameters. Collinearity relationships of *HD-Zip* genes among flax and other selected species were analyzed and visualized using TBtools software (v2.146) [25,26]. For each duplicated gene pair, the rates of nonsynonymous (Ka) and synonymous (Ks) substitutions per site were calculated using TBtools. The Ka/Ks ratio was subsequently derived to evaluate the pattern and strength of selective pressure acting on these gene pairs. The divergence time (million years ago, Mya) for each gene pair was estimated using the following formula, based on a synonymous substitution rate of 6.1 × 10^−9^ substitutions per site per year [27]:

Mya = Ks/(2 × 6.1 × 10^−9^) × 10^−6^.

### 2.4. Gene Structure and Promoter Analysis

The exon–intron structures of *LuHD-Zip* genes were determined based on alignments of genomic sequences and coding sequences (CDS). These structures were visualized using the Gene Structure Display Server (GSDS; http://gsds.cbi.pku.edu.cn/, accessed on 26 December 2025) [28]. For promoter analysis, the 2000 bp upstream sequences from the start codon of each *LuHD-Zip* gene were extracted from the “Longya-10” genome. Putative cis-acting regulatory elements within these promoter regions were identified using the PlantCARE database (http://bioinformatics.psb.ugent.be/webtools/plantcare/html/, accessed on 26 December 2025) [29].

### 2.5. Phylogenetic Analysis

We performed a phylogenetic analysis using HD-zip protein sequences from rice [9], maize [21], *Arabidopsis* [6], soybean [12], and potato [10]. Multiple sequence alignment was conducted with the ClustalW module in MEGA (v. 12.0) under default settings, which was then used to construct a phylogenetic tree by the neighbor-joining method (Poisson model, pairwise deletion). The tree was evaluated with 1000 bootstrap replicates and visualized in R (version 4.1.3).

### 2.6. Transcription Factor Binding Site Prediction

To identify the binding sites of HD-Zip transcription factors across the entire flax genome, the 2000 bp upstream regions of flax genes were retrieved using the RSAT Plants software [30]. The position weight matrices (PWMs) of *Arabidopsis* HD-Zip transcription factors were obtained from the JASPAR CORE database in MEME format [31]. The upstream region of each gene was scanned against the PWMs to identify HD-Zip transcription factor binding sites using the Find Individual Motif Occurrences (FIMO, version 5.5.9) tool, with a *p*-value threshold of less than 10^−6^. Sequences corresponding to each HD-Zip motif obtained from the JASPAR database were scanned using the FIMO tool [32].

### 2.7. Protein–Protein Interaction Analysis

The protein–protein interaction (PPI) network of flax *HD-Zip* genes from the perspective of A. thaliana homologous unigenes was constructed using the STRING (version 11.0) tool [33].

### 2.8. Gene Expression Analysis

Total RNA was extracted from frozen tissue samples using the EZgene Plant Easy Spin RNA Miniprep Kit (BIOMIGA, San Diego, CA, USA) following the manufacturer’s protocol. First-strand cDNA synthesis was performed with the PrimeScript RT Reagent Kit including gDNA Eraser (Takara, San Jose, CA, USA) according to the manufacturer’s instructions. Gene-specific primers for quantitative real-time PCR (qRT-PCR) were designed using Primer Premier 5.0 software (PREMIER Biosoft International, Palo Alto, CA, USA). Due to high sequence similarity among certain gene pairs (e.g., *LuHD-Zip19/31*, *LuHD-Zip13/22*, *LuHD-Zip21/7*, *LuHD-Zip15/26*, and *LuHD-Zip18/2*), it was not feasible to design specific primers for each individual gene. Consequently, a single primer pair was designed to amplify both members of each gene pair. All primer sequences used in this study are listed in Table S7. The qRT-PCR assays were performed on an Eco Real-Time PCR System (Illumina, San Diego, CA, USA) using the TB Green^®^ Premix Ex Taq™ II (TaKaRa, Dalian, China) as described by Qi et al. (2023) [34]. The thermal cycling conditions were as follows: initial denaturation at 95 °C for 30 s, followed by 40 cycles of denaturation at 95 °C for 5 s and annealing/extension at 60 °C for 30 s. A melting curve analysis was performed at the end of the PCR program (95 °C for 15 s, 60 °C for 1 min, and 95 °C for 15 s) to verify the specificity of the amplification products. The *GAPDH* gene was used as the internal reference for normalization [2]. Relative expression levels of *LuHD-Zip* genes were calculated using the 2^−∆∆Ct^ method and presented as the mean ± standard deviation (SD) from three independent biological replicates, each with three technical replicates. For data normalization and visualization, expression values were log_10_-transformed, and samples harvested at 0 h served as the calibrator for each treatment [35].

## 3. Results

### 3.1. Identification and Physicochemical Properties of HD-Zip Genes in Flax

A genome-wide search of the flax genome identified 34 *HD-Zip* genes. Based on their chromosomal distribution, these genes were systematically designated *LuHD-Zip1* to *LuHD-Zip34* (Table S1). Analysis of their deduced amino acid sequences revealed considerable variation in protein length, ranging from 200 (*LuHD-Zip15*) to 372 (*LuHD-Zip33*) amino acids. Accordingly, the predicted molecular weights varied between 22.65 kDa (*LuHD-Zip26*) and 40.34 kDa (*LuHD-Zip33*), with an average of approximately 31.85 kDa. The theoretical isoelectric points (pI) of the deduced LuHD-Zip proteins ranged from 4.49 to 9.46, with the majority (20 out of 34 members) exhibiting pI values below 7, indicating a preponderance of acidic proteins within this family. All identified HD-Zip proteins displayed grand average of hydropathicity (GRAVY) values below zero (ranging from −1.143 to −0.461), suggesting that they are predominantly hydrophilic. Furthermore, subcellular localization predictions consistently assigned all 34 LuHD-Zip proteins to the nucleus, supporting their putative role as transcription factors. The complete amino acid sequences of the LuHD-Zip proteins are provided in Table S2.

### 3.2. Phylogenetic Analysis of LuHD-Zip Proteins

**Figure 1:**
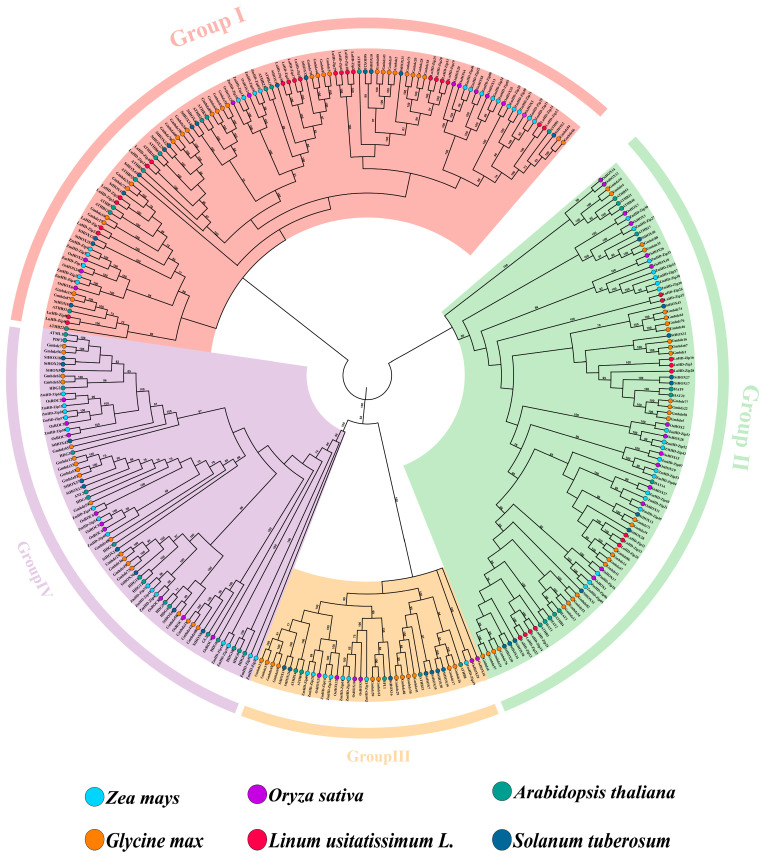
Phylogenetic tree of HD-Zip proteins from Longya-10, rice, maize, *Arabidopsis*, soybean, and potato. The tree is divided into four clades, each represented by a distinct color and designated as Group I to Group IV. Colored dots indicate the HD-Zip members from different species.

In this study, a total of 313 HD-Zip protein sequences were identified across six plant species, namely flax, rice (*Oryza sativa*), maize *(Zea mays*), *Arabidopsis thaliana*, soybean (*Glycine max*), and potato (*Solanum tuberosum*). Among them, the *LuHD-Zip* gene family comprised 34 members (Table S3). To elucidate the evolutionary relationships among these proteins, a phylogenetic tree was constructed using the neighbor-joining (NJ) method (Figure 1. Phylogenetic tree of HD-Zip proteins from Longya-10, rice, maize, Arabidopsis, soybean, and potato. The tree is divided into four clades, each represented by a distinct color and designated as Group I to Group IV. Colored dots indicate the HD-Zip members from different species. The results revealed that all identified *HD-Zip* genes were clustered into two distinct subfamilies, designated HD-Zip I and HD-Zip II. Notably, the distribution of genes within each subfamily varied considerably among species. Specifically, flax possessed a significantly higher number of HD-Zip I genes than *Arabidopsis*, whereas potato, a dicotyledonous species, harbored the fewest HD-Zip II genes among the dicots examined.

### 3.3. Conserved Motifs, Domains, and Gene Structure of LuHD-Zip Family Members

**Figure 2:**
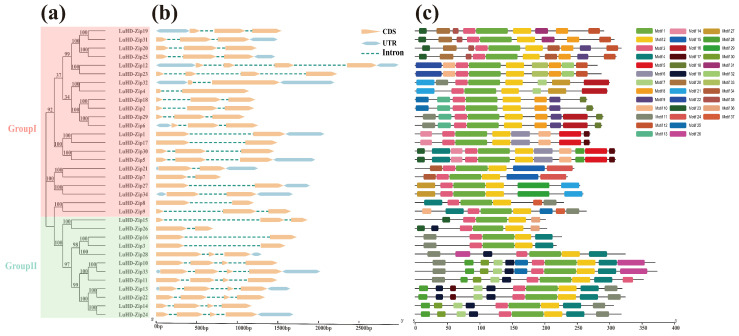
Phylogenetic relationships, gene structure, and conserved motifs of *LuHD-zip* genes. (**a**) Phylogenetic tree of LuHD-zip proteins. (**b**) Gene structure of *LuHD-zip* genes. (**c**) Motif composition of LuHD-zip proteins identified using MEME. Different colors represent distinct motifs.

To characterize the structural features of the LuHD-Zip family, we integrated analyses of phylogenetic relationships, conserved motifs, and exon-intron organization. A Neighbor-Joining phylogenetic tree was constructed based on the full-length amino acid sequences of all 34 LuHD-Zip proteins, which resolved the family into two major clades, designated as Group I and Group II (Figure 2a).

Analysis of the exon-intron organization revealed considerable variation in gene length among *LuHD-Zip* members, ranging from 705 bp (*LuHD-Zip26*) to 2991 bp (*LuHD-Zip12*) (Figure 2b; Table S1). The number of exons per gene ranged from two to four. Specifically, 12 members contained two exons, 13 members possessed three exons, and the remaining nine members harbored four exons. Notably, this exon number distribution was comparable between Group I and Group II.

The conserved motif architecture of LuHD-Zip proteins was investigated using the MEME suite, which identified 37 distinct motifs (Motifs 1–37) (Figure 2c; Table S4). Based on their distribution patterns across the two phylogenetic groups, these motifs were categorized into three types: (i) ubiquitous motifs present in all LuHD-Zip members; (ii) group-specific motifs restricted to either Group I or Group II; and (iii) shared motifs present in members of both groups. Among these, Motifs 1, 2, and 3 were identified as ubiquitous motifs, collectively constituting the core structural framework of HD-Zip proteins. Group I-specific motifs included Motifs 5, 6, 8–10, 12–14, 16, 18, 20–24, 27, 29, and 31–37, whereas Motifs 26 and 30 were uniquely present in Group II. Additionally, eight motifs (Motifs 4, 7, 11, 15, 17, 19, 25, and 28) were shared between members of both groups.

### 3.4. Chromosomal Distribution and Syntenic Analysis

**Figure 3:**
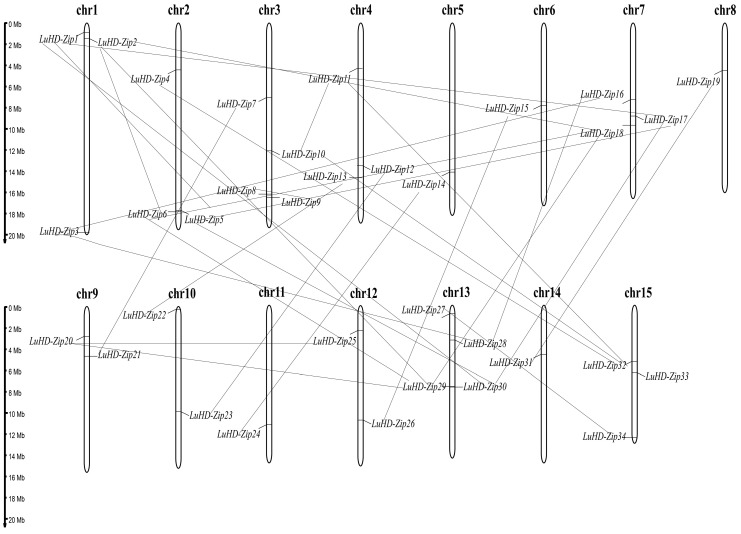
Chromosomal distribution of *LuHD-Zip* genes. Gray lines represent gene pairs that underwent segmental duplication.

The chromosomal distribution of the 34 identified *LuHD-Zip* genes was mapped onto the 15 flax chromosomes (Figure 3). The results revealed an uneven distribution pattern across chromosomes. Chromosomes 3 and 13 contained the highest number of *LuHD-Zip* genes, each harboring four members, whereas chromosomes 5, 6, 8, 11, and 14 each carried only a single gene. Chromosomes 9, 10, and 12 each contained two genes, while chromosomes 1, 2, 4, 7, and 15 each possessed three genes.

To investigate the expansion mechanisms of the *LuHD-Zip* gene family, we analyzed duplication events among the identified members. A total of 29 segmental duplication (SD) events were identified, involving all 34 *LuHD-Zip* genes (Table 1). Notably, no tandem duplication (TD) events were detected, suggesting that segmental duplication has been the primary driver of LuHD-Zip family expansion in flax. To assess the evolutionary constraints acting on duplicated gene pairs, we calculated the nonsynonymous substitution rate (Ka), synonymous substitution rate (Ks), and their ratio (Ka/Ks) for each SD pair. All 29 duplicated gene pairs exhibited Ka/Ks ratios less than 1, with values ranging from 0.0456 to 0.5009 (Table S5). This indicates that these gene pairs have evolved under strong purifying (negative) selection, suggesting functional constraints during their evolutionary history.

**Figure 4:**
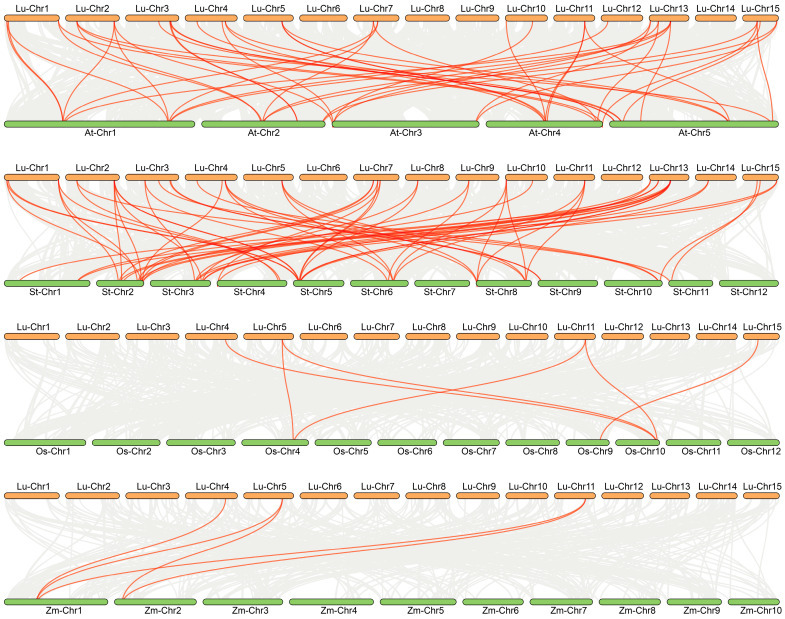
Syntenic analysis of *HD-Zip* genes between flax and four representative plant species (*Arabidopsis*, potato, rice, and maize). Gray lines in the background represent collinear blocks within and between genomes, while colored lines highlight syntenic *HD-Zip* gene pairs.

To explore the evolutionary relationships of *HD-Zip* genes across different species, we performed comparative syntenic analysis between flax and four representative plant species, including two dicotyledons *(Arabidopsis thaliana* and potato) and two monocotyledons (rice and maize) (Figure 4; Table S5) A total of 47 collinear gene pairs were identified between flax and *Arabidopsis*, while 60 pairs were detected between flax and potato. In contrast, only six and five collinear gene pairs were observed between flax and rice, and between flax and maize, respectively. These results indicate a substantially higher degree of syntenic conservation between flax and dicotyledonous species compared to monocotyledons. The collinear relationships were predominantly one-to-many or many-to-one. For instance, *LuHD-Zip30* exhibited collinearity with four potato *HD-Zip* genes, reflecting complex evolutionary relationships. Notably, three flax genes (*LuHD-Zip13*, *LuHD-Zip14*, and *LuHD-Zip24*) displayed collinear counterparts in all four examined species, suggesting their potential origin from an ancestral genomic region conserved across both dicotyledonous and monocotyledonous lineages. Conversely, three members (*LuHD-Zip15*, *LuHD-Zip20*, and *LuHD-Zip26)* lacked collinear orthologs in any of the four species, indicating lineage-specific evolution or potential gene loss in these lineages.

### 3.5. Cis-Acting Elements on LuHD-Zip Promoters

**Figure 5:**
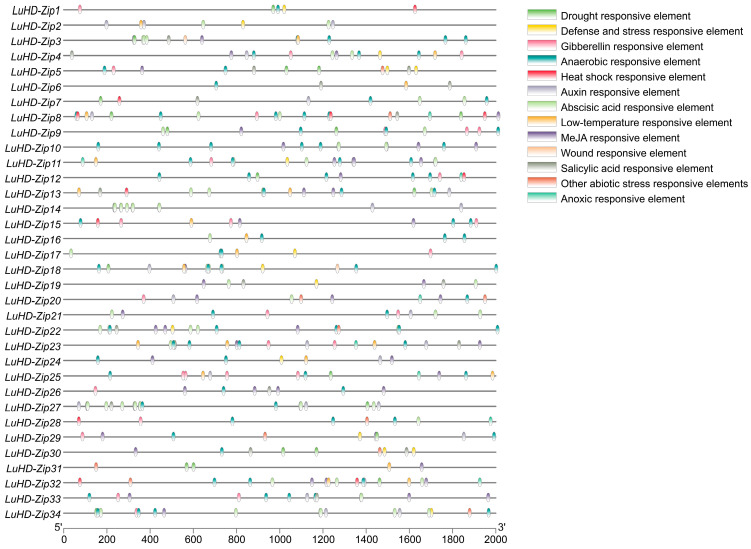
Distribution of *cis*−acting elements in the promoters of *LuHD-Zip* genes. Different colored boxes represent distinct *cis*−acting elements.

To comprehensively investigate the transcriptional regulatory mechanisms and potential biological functions of *LuHD-Zip* genes, this study extracted the 2000 bp upstream non-coding sequences of each gene from the genome. Subsequently, a systematic analysis of the promoter regions was conducted using bioinformatics tools to predict and identify potential cis-acting elements (Figure 5 and Table S6). The analysis and identification revealed two major categories of cis-acting elements: those responsive to stress and those associated with plant hormones. These elements play a critical role in the modulation of gene expression, encompassing promoters responsible for transcription initiation, enhancers that amplify transcriptional efficiency, and regulatory sequences that react to environmental cues. Among these, Stress-responsive elements included drought-inducible elements (MBS), low-temperature response(LTR), heat shock-related elements (CCAAT-box), defense and stress responses (TC-rich repeats) and wound-responsive element (WUN-motif), with respective totals of 23, 21, 11, 15 and 2. Furthermore, a comprehensive identification of hormone-responsive elements was conducted, revealing the presence of gibberellin-responsive elements, auxin-responsive elements, salicylic acid-responsive elements, abscisic acid-responsive elements, and MeJA-responsive elements, with respective counts of 29, 24, 18, 76, and 102. Among the *LuHD-Zip* genes, *LuHD-Zip8* exhibited the highest diversity of cis-acting elements (11 types), while *LuHD-Zip14* had the fewest (2 types). These findings indicate that members of the *LuHD-Zip* gene family may participate in plant hormone signaling pathways, as well as responses to abiotic and biotic stresses.

### 3.6. Prediction of Transcription Factor Binding Sites

By analyzing the binding site characteristics of Arabidopsis HD-Zip motifs from the JASPAR CORE database, 52 unique HD-Zip motifs were identified. Examination of these HD-Zip motifs across the upstream regions of the flax genome revealed that, among the 52 profiles associated with Arabidopsis HD-Zip transcription factor binding sites, 51 exhibited statistically significant and variable frequency distributions in the flax genome. The highest frequency was observed for MA1327.1, characterized by a 22-nucleotide motif corresponding to 161 binding sites. A genome-wide scan for HD-Zip profiles in flax identified a total of 2431 HD-Zip transcription factor binding sites distributed across 2435 distinct genes. The number of binding sites per intergenic region ranged from one to four, as detailed in Supplementary Table S8.

### 3.7. Analysis of Protein–Protein Interactions

**Figure 6:**
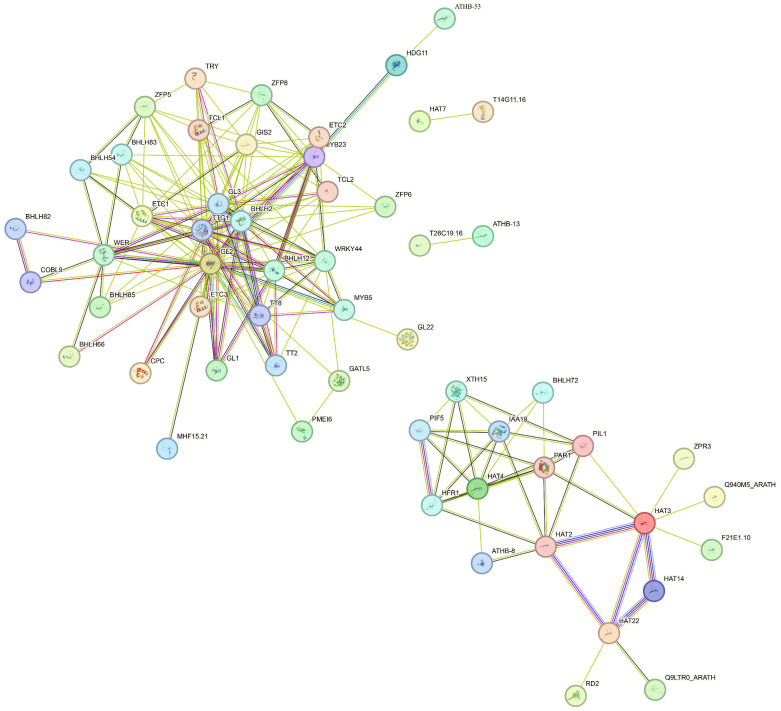
Protein–protein interaction (PPI) network of flax HD-Zip proteins predicted based on Arabidopsis thaliana orthologs.

To explore functional cooperation among flax HD-Zip proteins, a protein–protein interaction (PPI) network was constructed using the STRING database based on their orthologs in Arabidopsis thaliana, with a high confidence threshold (combined score ≥ 0.700). The resulting network exhibited a modular architecture, comprising a large primary core cluster in the upper-left region and a smaller secondary core cluster in the lower-right region. No isolated nodes were observed, suggesting widespread and evolutionarily conserved interactions among LuHD-Zip family members. Within the primary cluster, hub genes including GL2, TTG1, and ETC1 formed highly interconnected regulatory complexes with multiple HD-Zip proteins as well as other transcription factors such as bHLH and MYB family members, positioning them as critical nodes for network stability. The secondary cluster, centered around HAT2 and HAT3, appeared to be involved in more specialized regulatory processes. Collectively, these findings reveal both functional coordination and subfamily-level divergence within the flax HD-Zip family, providing a foundation for further investigation of their roles in abiotic stress responses (Figure 6).

### 3.8. Expression Analysis of LuHD-Zip Genes

**Figure 7:**
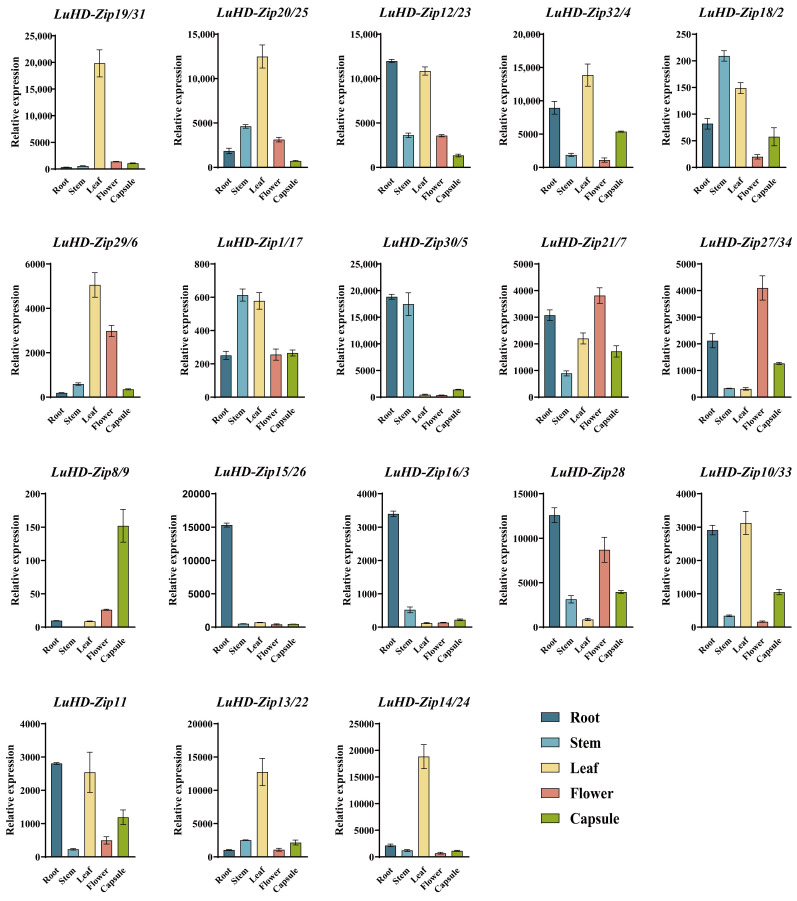
Tissue-specific expression profiles of *LuHD-Zip* genes in flax. Relative expression levels of 34 *LuHD-Zip* genes (classified into HD-Zip subfamilies I and II) were determined in root, stem, leaf, flower, and capsule tissues via qRT-PCR. The *x*-axis indicates the tested flax tissues, and the *y*-axis represents the relative expression level. Error bars denote the standard deviation of three biological replicates. Genes are labeled with their respective names and assigned HD-Zip subfamily classifications in parentheses.

To elucidate the tissue-specific functional divergence of HD-Zip transcription factors in flax, the relative expression levels of 34 *LuHD-Zip* genes were quantified across five distinct flax tissues (root, stem, leaf, flower, and capsule) using quantitative real-time PCR (qRT-PCR) (Figure 7). The expression profiles exhibited pronounced tissue-specificity, with distinct transcriptional patterns observed for genes belonging to HD-Zip subfamilies I and II. Within the HD-Zip subfamily I, *LuHD-Zip21/7* and *LuHD-Zip27/34* showed flower-specific enrichment, with their expression levels in flowers significantly surpassing those in roots, stems, leaves, and capsules. A suite of six subfamily II genes, including *LuHD-Zip15/26*, *LuHD-Zip16/3*, *LuHD-Zip28*, *LuHD-Zip11*, *LuHD-Zip13/22*, and *LuHD-Zip14/24*, exhibited root-dominant expression patterns, with the highest transcript levels recorded in roots. Notably, *LuHD-Zip8/9* was the only gene across all analyzed LuHD-Zip family members that displayed maximal expression in capsules, with its transcriptional abundance in this tissue being substantially higher than in other organs. For HD-Zip subfamily II members, *LuHD-Zip18/2* and *LuHD-Zip1/17* displayed the highest transcript accumulation in stems, with significantly lower expression detected in leaves, flowers, and capsules. In contrast, *LuHD-Zip19/31*, *LuHD-Zip20/25*, *LuHD-Zip32/4*, and *LuHD-Zip29/6* showed predominant expression in leaves, with their transcript levels being markedly higher in this tissue compared to roots, stems, flowers, and capsules. *LuHD-Zip12/23* and *LuHD-Zip30/5* exhibited root-preferential expression, with transcript abundances in roots exceeding those in all other tested tissues.

**Figure 8:**
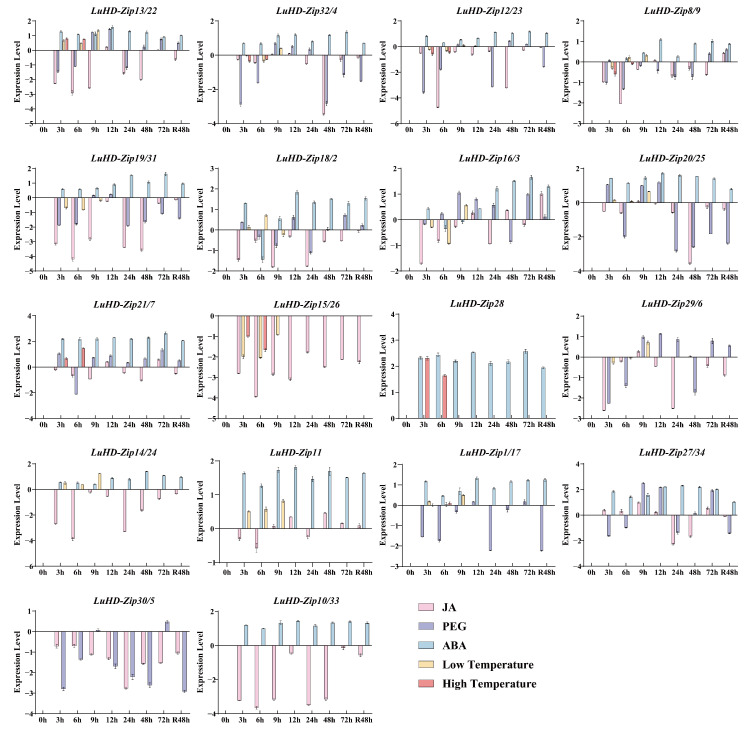
The expression levels of *LuHD-Zip* genes under different hormones. The *x*-axis represents the time points (0, 3, 6, 9, 12, 24, 48, and 72 h) after hormone treatment and 48 h after recovery in 1/2 MS liquid medium without hormone supplementation, while the *y*-axis indicates the gene expression levels. Relative expression levels were calculated using the 2^−∆∆Ct^ method, followed by logarithmic transformation (log10). All results were derived from three biological replicates, with error bars representing ± SD (*n* = 3).

**Figure 9:**
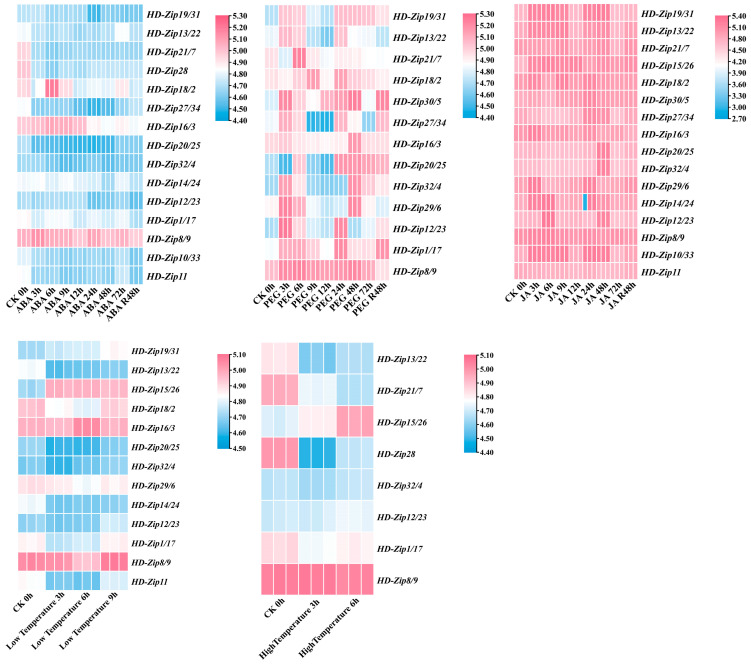
Heatmaps of *LuHD-Zip* gene expression under abiotic stresses. Log2-transformed fold changes relative to the control (0 h) are shown, with red indicating upregulation and blue indicating downregulation. The five panels correspond to ABA, PEG (drought), JA, low temperature, and high temperature treatments across multiple time points (3, 6, 9, 12, 24, 48, and 72 h, as well as 48 h recovery). Hierarchical clustering was applied to group genes with similar expression profiles.

To characterize the transcriptional responses of *LuHD-Zip* genes to abiotic stresses, qRT-PCR was performed under five treatments (ABA, PEG, JA, low temperature, and high temperature) at multiple time points. Expression profiles were visualized using bar charts and heatmaps based on log2-transformed fold changes relative to the control (CK 0 h) (Figure 8 and Figure 9). Overall, *LuHD-Zip* genes displayed highly divergent and stress-specific expression patterns, with no consistent response across treatments, indicative of functional diversification during stress adaptation. Under ABA exposure, most *LuHD-Zip* genes were continuously downregulated from 3 to 48 h, with *LuHD-Zip13/22*, *LuHD-Zip20/25*, *LuHD-Zip14/24*, and *LuHD-Zip10/33* showing the strongest repression (log2FC values as low as −4.40). Only a few genes, such as *LuHD-Zip18/2*, exhibited slight early upregulation, implying that most *LuHD-Zip* genes act as negative regulators in ABA-dependent signaling. PEG-simulated drought induced heterogeneous expression patterns. *LuHD-Zip16/3*, *LuHD-Zip20/25*, and *LuHD-Zip8/9* were strongly and stably upregulated (log2FC > 5.0), whereas *LuHD-Zip19/31* and *LuHD-Zip13/22* were markedly repressed. Other genes showed dynamic temporal fluctuations, suggesting multifaceted regulatory roles. JA treatment predominantly activated *LuHD-Zip* expression. The majority of *LuHD-Zip* genes, including *LuHD-Zip15/26*, *LuHD-Zip30/5*, *LuHD-Zip27/34*, and *LuHD-Zip11*, were persistently upregulated (log2FC up to 5.40), supporting their function as key components in JA-mediated stress and defense pathways. Under low temperature, *LuHD-Zip* genes displayed a bimodal pattern: a large group was downregulated, while *LuHD-Zip15/26*, *LuHD-Zip18/2*, and especially *LuHD-Zip8/9* (log2FC > 5.0) were strongly induced, revealing functional specialization in cold adaptation. High temperature also triggered divergent responses, with *LuHD-Zip13/22* and *LuHD-Zip15/26* downregulated, and *LuHD-Zip18/2*, *LuHD-Zip16/3*, and *LuHD-Zip8/9* prominently upregulated. Notably, *LuHD-Zip8/9* showed the most consistent induction across multiple stresses, suggesting a central role in thermotolerance and broader stress responses.

## 4. Discussion

The productivity and caliber of crops are profoundly affected by unfavorable environmental factors. However, in modern agriculture, utilizing TF tools to adjust protein levels has become a popular method for improving crop yield and quality [36]. HD-Zip proteins are transcription factors unique to plants characterized by a homeodomain and a leucine zipper motif [14]. A substantial body of evidence underscores the pivotal role of HD-Zip in orchestrating plant responses and adaptations to environmental fluctuations, encompassing variations in light, temperature, salinity, and stress conditions [15,37,38,39,40]. With the continuous advancement of molecular bioinformatics technology, the *HD-Zip* gene family has been extensively identified and characterized in a wide range of plant species, including *Arabidopsis thaliana* [7,8], rice (*Oryza sativa*) [9], potato [10], *Brassica rapa* [41], and maize (*Zea mays*) [42]. Nevertheless, the functional characterization of *HD-Zip* genes and their involvement in regulatory networks have not been systematically investigated in flax. Genome-wide identification of this gene family not only facilitates the elucidation of its genomic organization, structural characteristics, and evolutionary relationships among members, but also provides valuable candidate gene resources for subsequent functional studies.

In plants, HD-Zip proteins are generally classified into four subfamilies across most species: HD-Zip I, HD-Zip II, HD-Zip III, and HD-Zip I [6]. Phylogenetic analysis and sequence alignment reveal that *LuHD-Zip* genes are grouped into two subfamilies, consistent with findings reported in apple plants [43]. Arabidopsis subfamily III and IV genes are distinct and lack homologs in flax and apple species, indicating lineage-specific gene loss in these two plants. The HD-Zip I members in Arabidopsis exhibit greater diversity than HD-Zip II members, likely due to their earlier evolutionary origin, which allowed more time for gene duplication and rearrangement [44]. In flax, HD-Zip I is also the most abundant subfamily, aligning with previous reports.

The physicochemical characterization of the 34 HD-Zip proteins in flax unveiled comprehensive profiles, encompassing molecular weight, amino acid length, isoelectric point (pI), and hydrophobicity. The isoelectric point (pI) values of LuHD-Zip proteins span a range from 4.49 to 9.46, indicating their capacity to acquire varying net charges in response to environmental pH conditions. All LuHD-Zip proteins exhibit negative GRAVY (Grand Average of Hydropathy) values, reflecting their high hydrophilicity, which is a critical characteristic for membrane association and functional stability in cellular systems [45]. Furthermore, the subcellular localization analysis of flax HD-Zip proteins demonstrated their exclusive localization within the nucleus, a finding that is consistent with previous studies on HD-Zip proteins in other species [21,46]. Variations in gene structure are considered one of the representative traces of gene family evolution [47]. Analysis of gene architecture reveals a pattern that integrates evolutionary conservation with lineage-specific differentiation. The ubiquitous presence of motifs 1–3 across all LuHD-Zip members confirms their critical and indispensable role in homeodomain-mediated DNA binding and leucine zipper-mediated dimerization [48]. These motifs form a core structural framework that exhibits remarkable conservation throughout angiosperm evolution. In contrast, the identification of subfamily-specific motifs, exclusive to either subfamily I or II, provides molecular evidence for functional specialization between the two subfamilies—a finding consistent with the well-established functional divergence of HD-Zip I and II proteins observed in other species [49,50]. The exon–intron structure, consisting of 2–4 exons per gene, shows high similarity with that of Arabidopsis HD-Zip I/II [51] but differs from monocots such as rice, which typically possess longer introns and a higher number of exons [9]. This structural conservation within the flax subfamily, along with its divergence from monocots, not only supports the validity of the phylogenetic grouping but also reflects the broader evolutionary differentiation between eudicot and monocot lineages.

Regarding gene duplication, segmental duplications (rather than tandem duplications) have driven the expansion of this gene family. This study identified 29 pairs of segmentally duplicated genes, which reflects the complexity underlying the expansion of the *LuHD-Zip* gene family. The analysis of selection pressure on duplicated gene pairs according to three categories (purifying, positive, and neutral selection) serves as a valuable approach to elucidating their evolutionary history [52]. A Ka/Ks ratio less than 1 is interpreted as purifying selection, whereas a ratio equal to or greater than 1 suggests neutral or positive selection, respectively [27,53]. In this study, all duplicated *LuHD-Zip* gene pairs exhibited Ka/Ks ratios of less than 1. This finding not only confirms the predominant role of purifying selection throughout their evolutionary history but also supports the notion that these genes play important functions in growth and development.

The promoter regions of *HD-Zip* genes are enriched with hormone- and stress-responsive cis-elements, enabling them to perceive and integrate endogenous signals and exogenous environmental stimuli. Through this regulatory architecture, HD-Zip transcription factors modulate the expression of downstream target genes, thereby initiating specific physiological and biochemical processes that ultimately coordinate plant growth, development, and stress responses. As transcription factors, these genes not only regulate downstream gene expression but may themselves be subject to regulation by upstream factors or exert their functions through protein–protein interactions. Thus, the multilayered regulation of transcription factor activity, downstream gene expression, and protein interactions collectively constitutes a complex and interconnected regulatory network [54]. Accumulating evidence has demonstrated that HD-Zip transcription factors play pivotal roles in regulating plant responses to abiotic stress, thereby enhancing tolerance to multiple adverse environmental conditions. For instance, overexpression of *OsTF1L* in rice significantly enhances drought tolerance during vegetative growth by promoting photosynthetic efficiency and reducing water loss under drought stress [55]. Similarly, Zhang et al. reported that overexpression of a miR166-resistant version of *OsHB4* significantly improved drought tolerance in rice [56]. In maize, heterologous expression of the *AtHB6* also markedly enhanced drought tolerance in transgenic plants [57]. Furthermore, genes belonging to the HD-Zip I subfamily have been implicated in mitigating salt stress; for example, salt stress induces the expression and transcriptional activity of *AtHB1* in *Arabidopsis* [8]. In the present study, *LuHD-Zip* genes exhibited distinct tissue-specific expression patterns in flax, suggesting functional divergence among family members. Notably, *LuHD-Zip21/7* and *LuHD-Zip27/34* were highly and specifically expressed in flowers, reminiscent of the roles of AtHD-Zip III and IV subfamily members in Arabidopsis floral development [58,59]. These findings suggest that these genes may contribute to the morphogenesis of floral organs, such as petals and stamens/pistils, in flax. Conversely, *LuHD-Zip12/23* were predominantly expressed in roots, implying potential roles in root development or nutrient acquisition, while *LuHD-Zip19/31*, highly expressed in leaves, may be involved in leaf development or photosynthesis. The observed tissue-specific expression profiles provide a foundation for prioritizing candidate genes for functional validation using approaches such as in situ hybridization or tissue-specific gene silencing.

The stress-responsive expression profiles, visualized via log2FC-based heatmaps, revealed highly divergent and stress-specific responses, underscoring the functional diversification within the LuHD-Zip family. Under ABA exposure, the predominant downregulation of most *LuHD-Zip* genes suggests that many may act as negative regulators in ABA-dependent signaling pathways, potentially functioning as repressors that keep downstream stress-responsive genes silenced under non-stress conditions. This negative regulatory mode may serve to prevent unnecessary activation of stress responses under favorable growth conditions, thereby optimizing resource allocation. Conversely, under PEG-simulated drought stress, certain genes exhibited strong and stable upregulation, suggesting their positive regulatory roles in drought tolerance. The near-universal activation triggered by JA treatment strongly supports the involvement of *LuHD-Zip* genes in JA-mediated defense pathways [60], which is highly consistent with the enrichment of JA-responsive elements in their promoter regions. Under low temperature stress, a bimodal response pattern was observed, characterized by the strong induction of some genes and the repression of others. This expression divergence points to functional specialization within the family: certain members likely act as positive regulators of cold acclimation, while others may function as negative regulators whose suppression is required for the full activation of cold-responsive gene expression. This pattern aligns with previous observations in tomato [61]. Most notably, one gene (*LuHD-Zip8/9*) exhibited consistent and strong induction across multiple stresses (drought, low temperature, high temperature), suggesting it may function as a central integrator of diverse stress signals. Genes with such broad-spectrum stress responsiveness are relatively rare and often serve as master regulators or hub nodes in stress signaling networks. This makes *LuHD-Zip8/9* a particularly promising candidate for further functional validation, including overexpression and gene editing studies, and potential application in molecular breeding for broad-spectrum stress tolerance in flax.

This study provides the first systematic genome-wide characterization of the *HD-Zip* gene family in flax. The identified *LuHD-Zip* genes exhibit conserved structures, unique evolutionary patterns, and distinct tissue-specific and stress-responsive expression profiles. These findings extend our understanding of the HD-Zip family in fiber crops and provide promising candidate genes for further functional verification and molecular breeding aimed at improving stress resistance and fiber quality in flax.

Several methodological and conceptual limitations of this study should be acknowledged. First, the subcellular localization and transcriptional activity predictions are bioinformatic in nature and require experimental validation, for example via GFP fusion assays and transactivation studies in heterologous systems. Second, the precise molecular mechanisms by which *LuHD-Zip* genes modulate stress tolerance and development remain largely unknown; loss-of-function and gain-of-function approaches (e.g., CRISPR-Cas9 editing and stable overexpression) are urgently needed. Third, the upstream regulators and downstream target genes of LuHD-Zip proteins have not been experimentally identified; techniques such as yeast one-hybrid screening, ChIP-seq, and transcriptomic profiling of transgenic lines would be particularly valuable. Fourth, the use of single primer pairs for highly similar paralogs (e.g., *LuHD-Zip19/31*) limits the accurate quantification of individual gene expression; future studies will employ 3′ UTR-specific primers to resolve this issue and enable paralog-specific expression analysis.

## 5. Conclusions

This study presents the first genome-wide identification and comprehensive characterization of the *HD-Zip* gene family in flax, systematically revealing their evolutionary features, structural characteristics, and expression regulatory potential. A total of 34 *LuHD-Zip* genes were identified and phylogenetically classified into two subfamilies (I and II). Analyses of phylogenetic relationships, conserved domains, and motif compositions collectively demonstrated the high conservation of this family during plant evolution. Examination of gene structures and duplication events revealed that the LuHD-Zip family primarily expanded via segmental duplication and has undergone strong purifying selection, suggesting that these genes play important and stable roles in flax growth and development. Promoter cis-acting element analysis indicated that multiple *LuHD-Zip* genes harbor abundant hormone-responsive and stress-related regulatory elements, implying their potential involvement in regulating abiotic stress responses in flax. Furthermore, qRT-PCR results provided insights into the tissue-specific expression patterns and regulatory roles of *LuHD-Zip* genes under abiotic stress conditions. Additionally, phylogenetic and collinearity comparisons with dicotyledonous species (e.g., *Arabidopsis thaliana*, *Glycine max*, *Solanum tuberosum*) further supported the potential role of this gene family in modulating key agronomic traits such as fiber development and stress adaptation. Collectively, these findings not only provide a theoretical foundation for functional characterization of *HD-Zip* genes in flax but also establish candidate gene resources for their application in stress resistance breeding and fiber quality improvement.

## Figures and Tables

**Table 1:** Duplication events and divergence times of *LuHD-Zip* genes.

Seq1	Seq2	Duplication Event	Ka	Ks	Ka/Ks	Divergence Time (MYA)
*LuHD-Zip1*	*LuHD-Zip5*	SD	0.2204	1.4357	0.1535	117.680
*LuHD-Zip1*	*LuHD-Zip17*	SD	0.003	0.0627	0.0478	5.139
*LuHD-Zip1*	*LuHD-Zip30*	SD	0.2266	1.4769	0.1534	121.057
*LuHD-Zip2*	*LuHD-Zip6*	SD	0.2435	0.9798	0.2485	80.311
*LuHD-Zip2*	*LuHD-Zip18*	SD	0.0222	0.1408	0.1577	11.541
*LuHD-Zip2*	*LuHD-Zip29*	SD	0.2426	0.9061	0.2677	74.270
*LuHD-Zip3*	*LuHD-Zip16*	SD	0.0655	0.2843	0.2304	23.303
*LuHD-Zip3*	*LuHD-Zip28*	SD	0.3901	1.4056	0.2775	115.213
*LuHD-Zip5*	*LuHD-Zip17*	SD	0.2202	1.2394	0.1777	101.590
*LuHD-Zip6*	*LuHD-Zip18*	SD	0.2446	1.1012	0.2221	90.262
*LuHD-Zip7*	*LuHD-Zip21*	SD	0.0419	0.1247	0.3360	10.221
*LuHD-Zip8*	*LuHD-Zip9*	SD	0.1562	0.3371	0.4634	27.631
*LuHD-Zip10*	*LuHD-Zip11*	SD	0.1167	0.7823	0.1492	64.123
*LuHD-Zip22*	*LuHD-Zip13*	SD	0.0225	0.1336	0.1684	10.951
*LuHD-Zip23*	*LuHD-Zip12*	SD	0.0287	0.1393	0.2060	11.418
*LuHD-Zip24*	*LuHD-Zip14*	SD	0.0288	0.218	0.1321	17.869
*LuHD-Zip25*	*LuHD-Zip20*	SD	0.2146	0.443	0.4844	36.311
*LuHD-Zip26*	*LuHD-Zip15*	SD	0.0156	0.1065	0.1465	8.730
*LuHD-Zip27*	*LuHD-Zip34*	SD	0.0858	0.1713	0.5009	14.041
*LuHD-Zip28*	*LuHD-Zip16*	SD	0.4029	1.6713	0.2411	136.992
*LuHD-Zip29*	*LuHD-Zip6*	SD	0.0063	0.1382	0.0456	11.328
*LuHD-Zip29*	*LuHD-Zip18*	SD	0.2437	1.0143	0.2403	83.139
*LuHD-Zip29*	*LuHD-Zip20*	SD	0.5189	2.3748	0.2185	194.656
*LuHD-Zip30*	*LuHD-Zip5*	SD	0.0092	0.0237	0.3882	1.943
*LuHD-Zip30*	*LuHD-Zip17*	SD	0.2263	1.2711	0.1780	104.189
*LuHD-Zip31*	*LuHD-Zip19*	SD	0.0704	0.163	0.4319	13.361
*LuHD-Zip32*	*LuHD-Zip4*	SD	0.0141	0.1623	0.0869	13.303
*LuHD-Zip32*	*LuHD-Zip10*	SD	0.0368	0.1563	0.2354	12.811
*LuHD-Zip32*	*LuHD-Zip11*	SD	0.0819	0.9345	0.0876	76.598

Note: SD represents segmental duplication.

## Data Availability

The original contributions presented in this study are included in the article/Supplementary Material. Further inquiries can be directed to the corresponding author.

## Supplementary Materials

The following supporting information can be downloaded at: https://www.mdpi.com/article/10.3390/cimb48040402/s1.
